# Changes in Stimulant Use Over Time Among Men Who Have Sex with Men in Philadelphia, Baltimore, and Washington, DC, 2008–2023

**DOI:** 10.1007/s10461-026-05098-2

**Published:** 2026-03-31

**Authors:** Danielle German, Tanner Nassau, Karine Yenokyan, Xinyi Li, Sydney Bornstein, Kate Drezner, Taylor Hoj, Molly Gribbin, Colin Flynn, Kathleen A. Brady, Irene Kuo

**Affiliations:** 1 Department of Health, Behavior and Society, Johns Hopkins Bloomberg School of Public Health, Behavior and Society, 615 North Wolfe Street, Baltimore, MD USA; 2 Philadelphia Department of Public Health, Philadelphia, PA USA; 3 Department of Epidemiology, Johns Hopkins Bloomberg School of Public Health, Baltimore, MD USA; 4 District of Columbia Department of Health, Washington, DC USA; 5 Department of Epidemiology, George Washington University Milken Institute School of Public Health, Washington, DC USA; 6 Maryland Department of Health, Baltimore, MD USA

**Keywords:** MSM, Stimulants, Cocaine, Crack, Methamphetamine, Mid-Atlantic

## Abstract

Stimulant use among men who have sex with men (MSM) can contribute to HIV risk and care challenges. Monitoring and responding to local trends are critical for Ending the HIV Epidemic (EHE) initiatives. We assessed stimulant use patterns in Baltimore, Philadelphia, and Washington, DC from 2008 to 2023 using National HIV Behavioral Surveillance data. We collected cross-sectional data in each city using venue-based sampling in 2008, 2011, 2014, 2017, and 2023. We estimated average predicted probabilities of methamphetamine, powder cocaine, and crack cocaine use, and evaluated differences in prevalence of stimulant use overall and by race/ethnicity. In DC, stimulant use was stable or declined. Methamphetamine use remained stable overall (8%), with observed decreases among Hispanic MSM (13% to 4%). In Baltimore, methamphetamine use increased (2% to 5%) overall, while powder cocaine slightly declined overall (15% to 13%), with decreases among Black MSM but increases among White and Hispanic MSM. Crack cocaine decreased slightly (12% to 11%). In Philadelphia, methamphetamine use rose substantially (3% to 10%), with the largest increase among White MSM (3% to 19%). Powder cocaine use was highest in Philadelphia, reaching 41% among White MSM in 2023. Trends in stimulant use among MSM differed across these three EHE-funded cities. Philadelphia showed pronounced increases, particularly among White MSM, while DC and Baltimore exhibited more modest or stable patterns. Comparable local data sources are essential for identifying city- and subpopulation-specific trends to inform focused, equitable prevention strategies within EHE.

**Supplementary Information:**

The online version contains supplementary material available at https://doi.org/10.1007/s10461-026-05098-2.

## Resumen

El consumo de estimulantes entre hombres que tienen sexo con hombres (HSH) contribuye al riesgo de VIH y a desafíos en la atención médica. El monitoreo y la respuesta a las tendencias locales son fundamentales para las iniciativas de Acabar con la Epidemia del VIH (EHE, por sus siglas en inglés). Evaluamos los patrones de consumo de estimulantes en Baltimore, Philadelphia y Washington, DC de 2008 a 2023 utilizando datos de la Vigilancia Nacional del Comportamiento frente al VIH. Recopilamos datos transversales en cada ciudad utilizando muestreo basado en lugares en 2008, 2011, 2014, 2017 y 2023. Estimamos las probabilidades promedio predichas del consumo de metanfetamina, cocaína en polvo y crack, y evaluamos las diferencias en la prevalencia del consumo de estimulantes en general y por raza/etnicidad. En DC, el consumo de estimulantes se mantuvo estable o disminuyó. El consumo de metanfetamina se mantuvo estable en general (8%), con disminuciones observadas entre HSH hispanos (13% a 4%). En Baltimore, el consumo de metanfetamina aumentó (2% a 5%) en general, mientras que el consumo de cocaína en polvo disminuyó ligeramente en general (15% a 13%), con disminuciones entre HSH negros pero aumentos entre HSH blancos e hispanos. El crack disminuyó ligeramente (12% a 11%). En Philadelphia, el consumo de metanfetamina aumentó sustancialmente (3% a 10%), con el mayor incremento entre HSH blancos (3% a 19%). El consumo de cocaína en polvo fue más alto en Philadelpha, alcanzando el 41% entre HSH blancos en 2023. Las tendencias en el consumo de estimulantes entre HSH difirieron en estas tres ciudades financiadas por EHE. Philadelphia mostró aumentos pronunciados, particularmente entre HSH blancos, mientras que DC y Baltimore exhibieron patrones más modestos o estables. Fuentes de datos locales comparables son esenciales para identificar tendencias específicas por ciudad y subpoblación que informen estrategias de prevención enfocadas y equitativas dentro de EHE.

## Introduction

In 2019, the United States began planning for Ending the HIV Epidemic (EHE), with an ambitious goal to reduce new infections of HIV by 90% by 2030 [1, 2]. While important progress toward EHE goals has been made, it remains unlikely that the 2030 goal will be reached without changes to programs focused on HIV prevention and care [3]. Despite significant advances in antiretroviral therapy and increasing availability of pre-exposure prophylaxis (PrEP) for those at increased risk for acquiring HIV [4], access remains out of reach for many who experience socioeconomic barriers, lack trust in medical providers, and face intersecting stigma related to HIV, gender, sexual identity, and drug use [5].

Gay, bisexual, same gender loving, and other men who have sex with men (MSM) have been disproportionately affected by HIV since the beginning of the epidemic [6]. Substance use is higher among MSM compared to men who do not have sex with men [7] and associated with a variety of physical and mental health conditions through direct and indirect pathways [8]. Studies have documented considerable heterogeneity in substance use among MSM, finding that use behaviors differ across settings and activities that are contextually shaped by regional HIV trends and demographic characteristics [9].

Stimulant use in particular is higher among MSM compared to other populations [10]: National data show between 6 and 17% of MSM report methamphetamine and 16–20% report cocaine use in the past year [11]. MSM use stimulants in various contexts and for a variety of different reasons, including recreational use, pleasure, and to enhance sexual experiences (commonly referred to as chemsex) [12]. However, stimulant use disorder has recently been described as an urgent health crisis [13] due to its association with a range of long-term and serious medical concerns [14], especially cardiovascular complications [15], neuropsychiatric effects [16], and overdose risk—particularly in the context of polysubstance use and increasing risk of unintentional fentanyl exposure [17]. Stimulant use has also often been shown to be associated with increased HIV transmission risk when used in the context of a sexual encounter [18] and chemsex in particular has been shown to increase sexually transmitted infection and HIV risk due to its association with condomless anal sex [19]. Additionally, MSM who use stimulants often face social adversity and barriers [20] to culturally competent treatment [21] that can disrupt engagement with prevention and care services [22–24]. Longitudinal research shows heterogeneous patterns of substance use among MSM, yet relatively stable choice of substance over time [25]. It is important to understand how substance use behaviors change over time within specific geographic and cultural contexts as public health strategies may need to be tailored to shifting local patterns [8]. Comparisons of drug use across jurisdictions show that prevalence of specific substances also varies across different geographic areas [26–28]. Recent data among MSM in the US shows that stimulant and recreational drug use patterns vary considerably across regions, partly due to socioeconomic variation [29], and that use tends to be higher in EHE jurisdictions [9]. This highlights the need for localized understandings of stimulant use to enhance the relevance and appropriateness of focused public health interventions.

The National HIV Behavioral Surveillance [30] program is a Centers for Disease Control and Prevention (CDC) funded anonymous health survey and HIV testing program currently administered by 19 local health departments and collaborating partners across the country [31]. The NHBS program is critical to monitoring EHE goals by providing essential data on behaviors, prevention access, and service gaps among populations most affected by HIV, both over time and across settings [32]. Using NHBS data collected among MSM in 2008, 2011, 2014, 2017, and 2023, we sought to measure factors associated with stimulant use among MSM over time and across three geographically proximate Mid-Atlantic cities: Baltimore, Philadelphia, and Washington D.C.

Each of these three EHE priority jurisdictions were selected for this analysis due to their high HIV incidence rates and represent urban centers with distinct demographics, epidemiological profiles, drug markets [33–35], and public health infrastructure that may influence both HIV transmission dynamics and substance use patterns. By examining trends across these three Mid-Atlantic EHE cities over a 15-year period, we can better understand how regional factors may influence stimulant use patterns among MSM, ultimately informing more effective, locally-tailored prevention, care, and treatment strategies.

## Methods

### Study Design

Data from the NHBS MSM cycles from Philadelphia, Baltimore, and Washington, DC were used. Data included in this analysis are cross-sectional and were collected in 2008, 2011, 2014, 2017, and 2023. Although NHBS data collection cycles generally occurr every three years for a specific population, the 2020 MSM cycle was excluded from this analysis due to disruptions in study implementation from the COVID-19 lockdown.

NHBS sampling and recruitment methods for the MSM cycles have been described elsewhere [32, 36]. In brief, time location venue-based sampling was used to generate a representative sample of the population of MSM who attend venues at which MSM congregate following formative research in each of these data collection years. Interested participants were screened and eligible participants provided verbal consent for participation. Eligible participants were cisgender men who self-reported oral or anal sex with another man in the past 12 months, 18 years or older, spoke either English or Spanish, and lived within the local metropolitan statistical area.

Study participation included an interviewer-administered socio-behavioral survey assessing sexual and drug use behaviors, health status and service utilization, and other social and health characteristics as well as voluntary HIV testing. Remuneration for participation and HIV testing changed over time and varied across the three cities. By 2023, participants received between $50-$75 for the interview and $25-$75 for HIV testing across the three cities.

### Variables

Stimulant use variables assessed non-injection methamphetamine, powdered cocaine, and crack cocaine use in the past 12 months. Participants were first asked if they ever “shot up or injected any drugs other than those prescribed for you” and the last time they injected. Those who reported injection drug use in the past 12 months were asked which drugs they injected. Participants were then asked about times they “may have used drugs but did NOT inject them”. Those who reported any non-injection drug use in the past 12 months were asked how often they used each of a series of drugs (more than once a day, once a day, more than once a week, once a week or less, never). Questions for “methamphetamine, which includes meth, crystal meth, speed, or crank”, “crack cocaine, and “smoke or snort powder cocaine” were dichotomized for analysis to indicate any use of each drug in the past 12 months (yes, no). Composite variables for any non-injection drug use in the past 12 months (yes, no) and any non-injection or injection drug use in the past 12 months (yes, no) were also created.

Race/ethnicity combined responses to “Do you consider yourself to be of Hispanic, Latino/a, or Spanish origin?” and “Which racial group or groups do you consider yourself to be in? You may choose more than one” for a categorical variable indicating non-Hispanic Black, non-Hispanic White, Hispanic/Latine. Those who reported American Indian or Alaska native, Asian, Native Hawaiian or Other Pacific Islander were grouped with those reporting multiple races given small sample sizes.

Additional variables included age (< 30, + 30), income (<$20 000, $20 000-$49 999, $50 000+), education (HS/GED or less, some college, BA+), sexual identity (gay, bi/heterosexual), homelessness in the past 12 months (yes, no), self-reported HIV status (“Have you ever tested positive for HIV, that is, do you have HIV?”: positive, negative), type of the recruitment venue (bar/club, sex environment/street location/park, other), and federal poverty level calculated from income and number of dependents (above the poverty level, below the poverty level).

### Analysis

To examine the prevalence of each outcome across time and to evaluate the association between the stimulant use and potential demographic factors and socio-economic disparities among MSM most affected by HIV in each jurisdiction, we estimated a set of univariate logistic models for non-Hispanic Black, non-Hispanic White, and Hispanic/Latine participants in each location. We then compared marginal probabilities of each stimulant use in three cities by standardizing the total sample to each wave of data collection in each location. Using the coefficient estimates from the logistic models, where we controlled for potential confounders such as year of data collection, age, sexual identity, self-reported HIV-positive status, homelessness in the past 12 months, recruitment venue type, and the federal poverty level, we obtained the predicted marginal probabilities of stimulant use for each person in each NHBS cycle in each city. We compared the distributions of the average predicted probabilities using Kruskal-Wallis test. The bootstrap procedure was used to generate 500 bootstrap samples to construct 95% confidence intervals for the average predicted probability of each stimulant use for each year of the data collection sample in each location.

Further analysis was done using stratification by race and ethnicity (non-Hispanic Black, non-Hispanic White, Hispanic/Latine) to assess potential racial differences in stimulant use among MSM in three Mid-Atlantic cities. However, predicted probability analyses are reported only for non-Hispanic Black and non-Hispanic White MSM, as estimates were unstable among Hispanic participants due to small sample size. The analysis was done using SAS software 9.4 (SAS Institute Inc.).

### Human Subjects

A waiver of written consent was granted by the CDC and IRBs due to the sensitive nature of the data collected. Therefore, only verbal consent was required and provided by participants which was documented by study staff. The NHBS protocols and local materials were reviewed and approved by the Institutional Review Boards for each site, respectively: the DC Department of Health and the George Washington University; the City of Philadelphia Department of Public Health; the Johns Hopkins Bloomberg School of Public Health and the Maryland Department of Health.

## Results

Table 1 shows the descriptive and drug use characteristics of the total sample across all waves by race/ethnicity for each site. The majority were over age 30, except among Hispanic MSM in Philadelphia (43%). Most identified as gay in each city, and a higher proportion of participants identified as bisexual or heterosexual in Baltimore (29%) and Philadelphia (25%) compared to DC (14%). Socioeconomic status (education, income) was notably higher in DC compared to the other two cities and among White compared to Black and Hispanic MSM in each city. Similarly, the proportion under the poverty level and reporting unhoused status in the past year was much lower in DC compared to the other two cities and among White MSM compared to others. Two-thirds of Baltimore MSM were recruited from bars or clubs, compared to approximately 50% in DC and Philadelphia. Approximately 10% were recruited from sex environments or street/park locations in each city; this proportion was lower among White MSM compared to others in DC and Baltimore and lower among Hispanic MSM compared to others in Philadelphia. Self-reported HIV status was similar in all three cities, ranging from 15.5% in DC to 18.6% in Baltimore, three times as high among Black MSM compared to White MSM in DC and Philadelphia and twice as high in Baltimore, and 2–3 times as high among Hispanic MSM compared with White MSM in each city.

History of any injection drug use was highest in Baltimore (8%), followed by Philadelphia (5%) and DC (5%), and 2–3 times higher among White MSM compared to other groups in DC and Baltimore. Less than 3% overall reported injection drug use in the past year across all cities. Approximately half reported any non-injection drug use, with the highest proportion among White MSM in Baltimore (58%) and DC (56%) and among Hispanic MSM in Philadelphia (55%).

**Table 1:** Demographic characteristics of MSM NHBS participants in Washington DC, Baltimore, and Philadelphia by race/ethnicity, 2008–2023

	Washington, DC				Baltimore, MD				Philadelphia, PA			
	Black MSM (*n* = 739) *n* (%)	White MSM (*n* = 816) *n* (%)	Hispanic MSM (*n* = 243) *n* (%)	Total (*n* = 1798) *N* (%)	Black MSM (*n* = 1490) *n* (%)	White MSM (*n* = 410) *n* (%)	Hispanic MSM (*n* = 92) *n* (%)	Total (*n* = 1992) *N* (%)	Black MSM (*n* = 1,421) *n* (%)	White MSM (*n* = 755) *n* (%)	Hispanic MSM (*n* = 339) *n* (%)	Total (*n* = 2,515) *n* (%)
Age												
< 30	366 (45.5)	333 (37.3)	139 (51.7)	838 (42.6)	640 (43.0)	137 (33.4)	39 (42.4)	816 (41.0)	702 (49.4)	247 (32.7)	192 (56.6)	1141 (45.4)
30+	438 (54.5)	560 (62.7)	130 (48.3)	1128 (57.4)	850 (57.1)	273 (66.6)	53 (57.6)	1176 (59.0)	719 (50.6)	508 (67.3)	147 (43.4)	1374 (54.6)
Income												
<$20,000	173 (21.8)	74 (8.3)	40 (14.9)	287 (14.7)	748 (52.1)	85 (20.8)	30 (33.0)	863 (44.6)	713 (51.3)	187 (25.3)	139 (41.9)	1039 (42.2)
$20,000–49,999	233 (29.4)	152 (17.1)	88 (32.8)	473 (24.2)	447 (31.1)	130 (31.9)	30 (33.0)	607 (31.4)	455 (32.8)	291 (39.3)	126 (38.0)	872 (35.4)
$50,000+	388 (48.9)	664 (74.6)	140 (52.2)	1192 (61.1)	242 (16.8)	193 (47.3)	31 (34.1)	466 (24.1)	221 (15.9)	262 (35.4)	67 (20.2)	550 (22.4)
Poverty												
Above	654 (82.4)	830 (93.4)	234 (87.3)	1718 (88.1)	773 (53.7)	334 (81.9)	62 (68.1)	1169 (60.3)	763 (55.0)	608 (82.3)	216 (65.1)	1587 (64.5)
Below	140 (17.6)	59 (6.6)	34 (12.7)	233 (11.9)	666 (46.3)	74 (18.1)	29 (31.9)	769 (39.7)	625 (45.0)	131 (17.7)	116 (34.9)	872 (35.5)
Education												
HS/GED or Less	194 (24.1)	56 (6.3)	43 (16.0)	293 (14.9)	788 (52.9)	90 (22.0)	28 (30.4)	906 (45.5)	755 (53.2)	187 (24.8)	162 (47.8)	1104 (43.9)
Some college	266 (33.1)	108 (12.1)	71 (26.4)	445 (22.6)	470 (31.6)	126 (30.7)	24 (26.1)	620 (31.1)	451 (31.8)	176 (23.3)	99 (29.2)	726 (28.9)
BA+	344 (42.8)	729 (81.6)	155 (57.6)	1228 (62.5)	231 (15.5)	194 (47.3)	40 (43.5)	465 (23.4)	214 (15.1)	392 (51.9)	78 (23.0)	684 (27.2)
Sexual Identity												
Gay	608 (76.4)	835 (93.5)	233 (87.6)	1676 (85.7)	930 (62.7)	329 (80.4)	65 (70.7)	1324 (66.7)	1000 (71.0)	633 (84.1)	251 (75.2)	1884 (75.5)
Bi/Hetero/Other	188 (23.6)	58 (6.5)	33 (12.4)	279 (14.3)	553 (37.3)	80 (19.6)	27 (29.4)	660 (33.3)	408 (29.0)	120 (15.9)	83 (24.9)	611 (24.5)
Homeless, past 12 mos.	64 (8.0)	23 (2.6)	11 (4.1)	98 (5.0)	242 (16.3)	36 (8.8)	10 (10.9)	288 (14.5)	260 (18.3)	93 (12.3)	56 (16.5)	409 (16.3)
Venue type												
Bar/Club	457 (57.5)	418 (48.4)	115 (43.4)	990 (51.5)	983 (66.5)	300 (73.9)	62 (67.4)	1345 (68.1)	779 (54.8)	391 (51.8)	198 (58.4)	1368 (54.4)
Street/Sex Envir/Park	106 (13.3)	48 (5.6)	14 (5.3)	168 (8.7)	185 (12.5)	19 (4.7)	9 (9.8)	213 (10.8)	152 (10.7)	88 (11.7)	22 (6.5)	262 (10.4)
Other	232 (29.2)	398 (46.0)	136 (51.3)	766 (39.8)	310 (21.0)	87 (21.4)	21 (22.8)	418 (21.2)	490 (34.5)	276 (36.6)	119 (35.1)	885 (35.2)
HIV positive (self-report)	194 (24.1)	72 (8.1)	39 (14.5)	305 (15.5)	318 (21.5)	43 (10.5)	6 (6.6)	367 (18.6)	302 (21.3)	55 (7.4)	57 (17.0)	414 (16.6)
Inject drugs ever	32 (4.0)	47 (5.3)	15 (5.6)	94 (4.8)	92 (6.2)	56 (13.7)	6 (6.5)	154 (7.7)	41 (2.9)	68 (9.0)	14 (4.1)	123 (4.9)
Inject drugs past 12 mos.	17 (2.1)	21 (2.4)	6 (2.2)	44 (2.2)	29 (2.0)	24 (5.9)	1 (1.1)	54 (2.7)	12 (0.8)	40 (5.3)	9 (2.7)	61 (2.4)
Non-injection drugs past 12 mos.	420 (52.2)	497 (55.7)	138 (51.3)	1055 (53.7)	802 (53.9)	235 (57.6)	47 (51.7)	1084 (54.5)	728 (51.3)	322 (42.7)	186 (55.0)	1236 (49.2)
Non-injection OR injection drug use, past 12 mo.	422 (52.5)	497 (55.7)	139 (51.7)	1058 (53.8)	810 (54.4)	241 (58.9)	47 (51.7)	1098 (55.2)	732 (51.6)	332 (44.0)	188 (55.6)	1252 (49.8)

### Prevalence of Stimulant Use Over Time

**Fig. 1:**
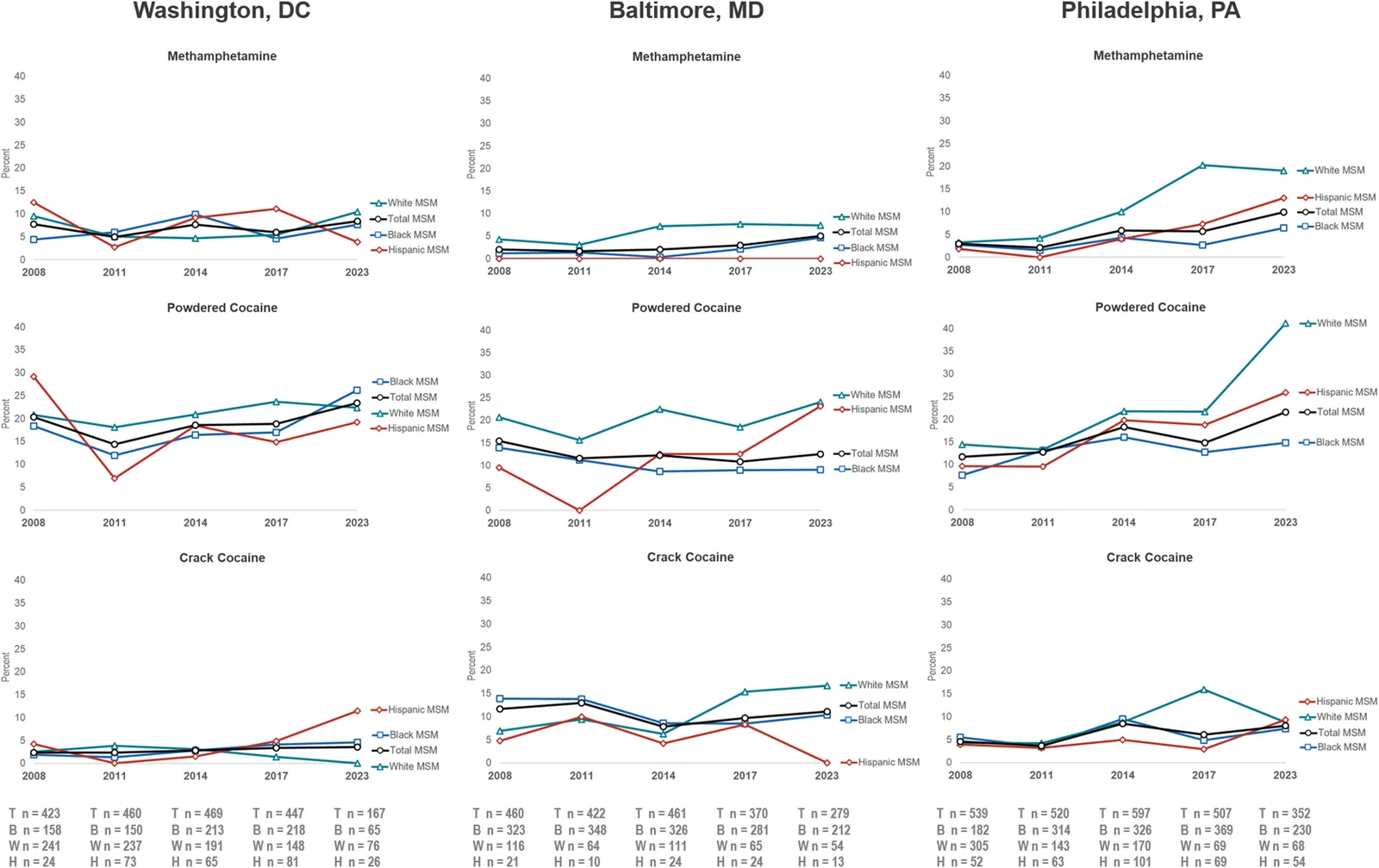
Stimulant use prevalence by race/ethnicity among MSM-NHBS participants in Washington, DC, Baltimore, and Philadelphia, 2008–2023

Figure 1 and supplemental Table 1 show the prevalence of past year methamphetamine, powder cocaine, and crack cocaine use for each city at each time point.

#### Methamphetamine

Between 2008 and 2023, overall prevalence of methamphetamine use increased from 3% to 10% in Philadelphia, 2% to 5% in Baltimore, and remained stable at 8% with some fluctuation over time in DC. Prevalence was highest among White MSM in DC in 2023 and in Baltimore and Philadelphia at each time point. The notably increased prevalence in Philadelphia occurred among all groups over time and was particularly striking among White MSM (3% to 19%) and Hispanic MSM (2% to 13%). Though prevalence was low among Black MSM, there were substantial increases in each city over time (3% to 7% in Philadelphia, 1% to 5% in Baltimore, 4% to 8% in DC). Smaller increases in prevalence occurred among White MSM in Baltimore (4% to 7%) and DC (10% to 11%). Prevalence decreased from 13% to 4% among Hispanic MSM in DC, although the sample size was relatively small.

#### Powdered Cocaine

Between 2008 and 2023, the overall prevalence of powdered cocaine use nearly doubled in Philadelphia (from 12% to 22%) in all groups, increased from 20% to 23% in DC, and decreased from 15% to 13% in Baltimore. In 2023, prevalence was highest among White MSM in Philadelphia and Baltimore and among Black MSM in DC. In DC, there was a larger increase among Black MSM (18% to 26%) compared to White MSM (21% to 22%) and a decrease among Hispanic MSM (29% to 19%) that may reflect sample size. In Baltimore, decreased prevalence was primarily among Black MSM (14% to 9%) while prevalence increased among White (21% to 24%) and the small proportion of Hispanic MSM (10% to 24%).

#### Crack Cocaine

Overall prevalence of crack cocaine use was highest in Baltimore, with relatively little change over the study period (12% to 11%). Prevalence increased in Philadelphia from 5% to 8% and in DC from 2% to 4%. Prevalence was highest among White MSM in Baltimore and among Hispanic MSM in DC and Philadelphia. Prevalence increased among all groups in Philadelphia, and only among Black (2% to 5%) and Hispanic MSM (2% to 4%) in DC. In Baltimore, crack use decreased among Black MSM (14% to 10%) and more than doubled among White MSM (7% to 17%).

### Odds and Correlates of Stimulant Use

Table 2 shows results of multivariate logistic regression models adjusted for sociodemographic characteristics, venue type and year on each drug type for each city, stratified by race.

**Table 2:** Multivariate analysis of past year stimulant use by type of drug among MSM in NHBS in Washington, DC, Baltimore, and Philadelphia over time, 2008–2023, stratified by race

	Washington, DC			Baltimore, MD			Philadelphia, PA		
	Black MSM (*n* = 739) OR (95% CI)	White MSM (*n* = 816) OR (95% CI)	Hispanic MSM (*n* = 243) OR (95% CI)	Black MSM (*n* = 1490) OR (95% CI)	White MSM (*n* = 410) OR (95% CI)	Hispanic MSM (*n* = 92) OR (95% CI)	Black MSM (*n* = 1421) OR (95% CI)	White MSM (*n* = 685) OR (95% CI)	Hispanic MSM (*n* = 256) OR (95% CI)
Methamphetamine									
Age 30+	1.04 (0.53, 2.02)	1.53 (0.74, 3.18)	1.70 (0.60, 4.82)	3.02 (0.96, 9.52)	0.72 (0.27, 1.88)	-	1.00 (0.52–1.90)	1.23 (0.65–2.32)	1.02 (0.32–3.25)
Identify as gay	1.04 (0.49, 2.20)	2.65 (0.63, 11.12)	3.09 (0.34, 27.99)	2.58 (0.97, 6.85)	1.08 (0.31, 3.79)	-	0.64 (0.33–1.27)	**0.31 (0.16–0.60)**	0.61 (0.18–2.07)
Below poverty level	1.03 (0.48,2.23)	**4.31 (1.75**,** 10.65)**	0.35 (0.03, 3.98)	1.15 (0.44, 3.02)	0.45 (0.11, 1.77)	-	0.68 (0.34–1.35)	1.02 (0.50–2.05)	2.08 (0.64–6.79)
Homeless,past 12 mos.	**7.21 (3.29**,** 15.82)**	**8.37 (2.61**,** 26.86)**	7.16 (0.78, 65.55)	**7.05 (2.63**,** 18.90)**	**4.45 (1.04**,** 19.12)**	-	**5.11 (2.54–10.28)**	**2.16 (1.06–4.41)**	**4.90 (1.61–14.89)**
HIV positive (self-report)	**4.21 (2.24**,** 7.92)**	**11.28 (5.73**,** 22.19)**	**3.12 (1.02**,** 9.59)**	1.08 (0.41, 2.85)	**4.91 (1.70**,** 14.19)**	-	**3.15 (1.64–6.06)**	**3.47 (1.54–7.80)**	1.40 (0.42–4.63)
Powdered cocaine									
Age 30+	1.32 (0.87, 1.99)	**0.53 (0.38**,** 0.76)**	0.61 (0.29, 1.28)	**1.49 (1.02**,** 2.18)**	1.10 (0.64, 1.88)	0.67 (0.15, 3.05)	1.34 (0.96–1.88)	0.85 (0.56–1.29)	1.08 (0.57–2.04)
Identify as gay	0.84 (0.4, 1.31)	2.25 (0.97, 5.21)	2.00 (0.60, 6.65)	1.02 (0.70, 1.49)	0.60 (0.32, 1.13)	5.09 (0.55, 46.66)	1.15 (0.79–1.68)	**0.59 (0.36–0.97)**	0.74 (0.37–1.45)
Below poverty level	0.98 (0.58, 1.67)	1.42 (0.75, 2.68)	1.38 (0.50, 3.80)	1.36 (0.94, 1.98)	1.17 (0.60, 2.28)	0.47 (0.07, 3.33)	1.36 (0.96–1.93)	1.04 (0.63–1.72)	0.72 (0.36–1.45)
Homeless,past 12 mos.	**2.53 (1.36**,** 4.71)**	2.62 (1.03, 6.68)	**5.70 (1.32**,** 24.56)**	**2.17 (1.43**,** 3.29)**	1.76 (0.74, 4.21)	8.30 (0.71, 97.66)	**2.64 (1.80–3.87)**	**1.74 (1.00-3.04)**	1.75 (0.80–3.83)
HIV positive (self-report)	1.02 (0.65, 1.60)	1.60 (0.88, 2.90)	0.37 (0.10, 1.42)	0.76 (0.47, 1.22)	0.98 (0.42, 2.29)	< 0.001 (< 0.001, > 999.99)	**2.64 (1.80–3.87)**	1.44 (0.72–2.87)	1.17 (0.54–2.53)
Crack cocaine									
Age 30+	1.78 (0.61, 5.23)	1.65 (0.55, 4.91)	0.26 (0.05, 1.49)	**6.15 (3.65**,** 10.37)**	1.26 (0.52, 3.04)	28.69 (0.39, > 999.99)	**6.11 (3.09–12.06)**	1.16 (0.55–2.46)	2.94 (0.88–9.79)
Identify as gay	0.91 (0.31, 2.67)	0.31 (0.09, 1.08)	3.51 (0.17, 73.42)	**0.53 (0.36**,** 0.79)**	**0.28 (0.12**,** 0.64)**	0.08 (0.00, 1.55)	**0.49 (0.29–0.85)**	**0.18 (0.08–0.38)**	1.05 (0.31–3.60)
Below poverty level	**4.49 (1.60**,** 12.58)**	**3.69 (1.13**,** 12.03)**	2.70 (0.44, 16.39)	**1.55 (1.03**,** 2.32)**	2.08 (0.88, 4.93)	12.24 (0.40, 374.41)	**3.27 (1.75–6.10)**	**4.10 (2.01–8.35)**	**6.33 (1.67–24.05)**
Homeless, past 12 mos.	**4.14 (1.39**,** 12.36)**	**13.38 (3.61**,** 49.54)**	52.39 (2.99, 917.04)	**4.54 (3.03**,** 6.79)**	**5.18 (1.90**,** 14.13)**	2.87 (0.10, 79.52)	**3.28 (1.93–5.56)**	**4.09 (1.96–8.56)**	2.97 (0.94–9.41)
HIV positive (self-report)	**3.96 (1.49**,** 10.52)**	**3.96 (1.23**,** 12.78)**	0.32 (0.02, 6.0)	0.99 (0.61, 1.61)	**3.09 (1.06**,** 8.98)**	23.33 (0.41, > 999.99)	1.37 (0.77–2.42)	2.74 (0.98–7.64)	0.97 (0.26–3.67)

All models adjusted for survey year (2008, 2011, 2014, 2017, 2023) and venue type (bar/club, sex environment/street location/park, other). Bold indicates statistically significant associations.

####  Methamphetamine

Adjusted odds of methamphetamine use were significantly greater among those reporting unhoused status in the past year among all groups in each city, except among Hispanic MSM in DC. The adjusted odds were also substantially greater for those reporting HIV-positive status among all MSM in DC, Black and White MSM in Philadelphia, and White MSM in Baltimore. The adjusted odds of methamphetamine use were also greater among White MSM below the poverty level in DC and lower among those identifying as gay in Philadelphia.

#### Powdered Cocaine

Adjusted odds of powdered cocaine use were substantially greater among those who were unhoused among Black MSM in all cities, among White MSM in Philadelphia, and Hispanic MSM in DC. Adjusted odds were greater among Black MSM reporting HIV-positive status in Philadelphia and older Black MSM in Baltimore. Adjusted odds were lower among younger White MSM in DC and those who identified as gay among White MSM in Philadelphia.

#### Crack Cocaine

The adjusted odds of crack cocaine use were substantially greater among those who were unhoused in the past year for Black and White MSM in all cities. The adjusted odds were substantially greater among Black MSM under the poverty level in all cities, White MSM in Philadelphia and DC, and Hispanic MSM in Philadelphia. Adjusted odds were substantially greater among older Black MSM in Baltimore and Philadelphia, HIV-positive White MSM in Baltimore, and HIV-positive Black and White MSM in DC. Adjusted odds decreased among Black and White MSM who identified as gay in Baltimore and Philadelphia.

### Average Predicted Probability of Stimulant Use

#### Methamphetamine

**Fig. 2:**
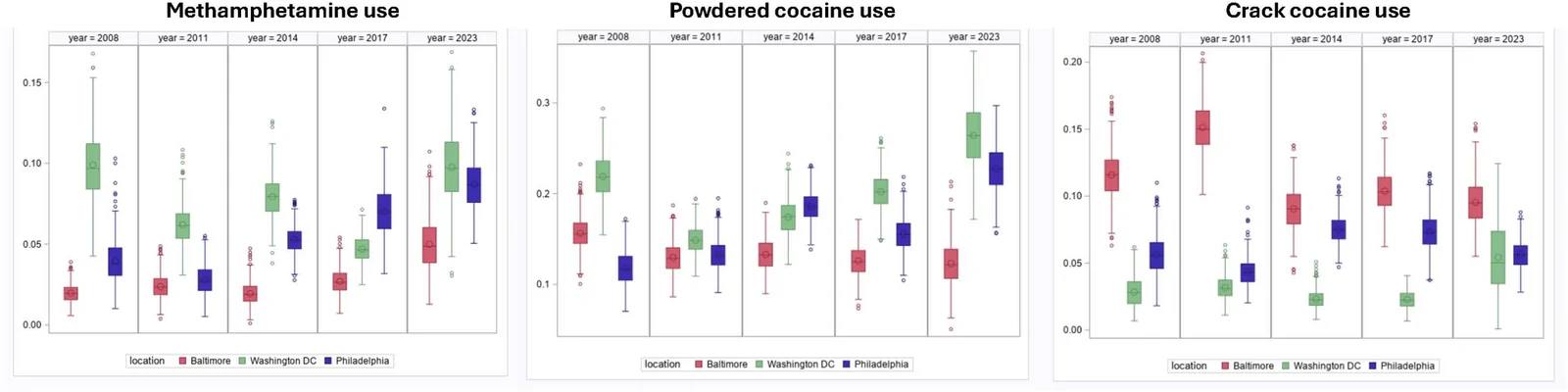
Distribution of the average predicted probability of stimulant use among MSM-NHBS participants in Washington, DC, Baltimore, and Philadelphia, 2008–2023

**Fig. 3:**
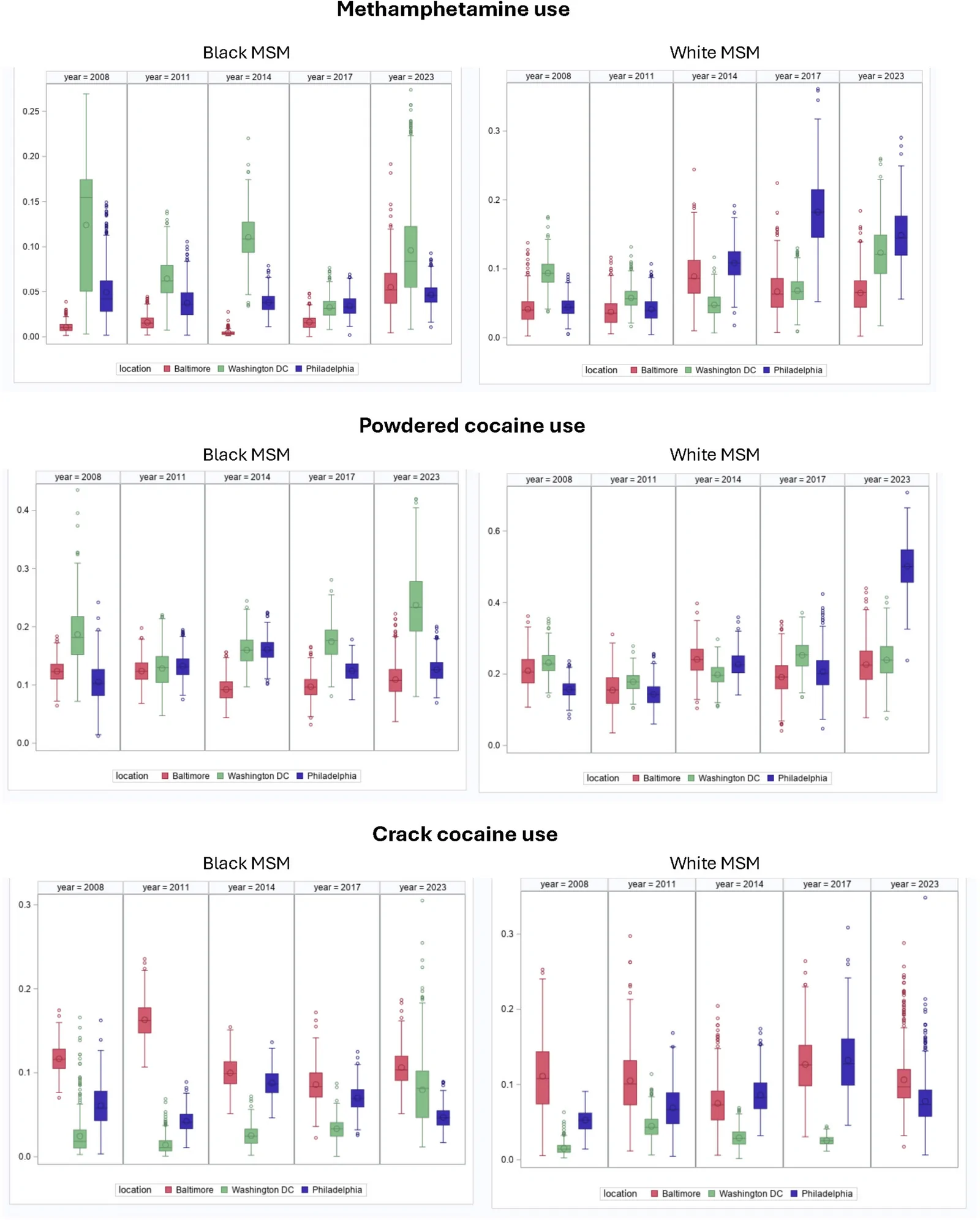
Distribution of the average predicted probability of stimulant use by race/ethnicity among MSM-NHBS participants in Washington, DC, Baltimore, and Philadelphia, 2008–2023

Accounting for the demographic characteristics of the samples at each time point (Figs. 2 and 3 and Supplemental Tables 2 & 3), the overall average predicted probability (APP) of methamphetamine use remained stable at 1% in DC (2008–2023 comparison non-significant), increased from 2% to 5% in Baltimore, and increased from 4% to 9% in Philadelphia. Among Black MSM, APP decreased from 17% to 5% in DC but increased in Baltimore (1% to 5%) and Philadelphia (4% to 5%, though the overall change was not statistically significant). Among White MSM, APP increased in all cities: DC (9% to 13%), Baltimore (4% to 7%), and Philadelphia (4% to 15%). Pairwise comparisons (Supplemental Table 4) confirm statistically significant changes over time for most trends. There was notable stability in DC use overall, among Black MSM in Philadelphia during multiple intermediate periods and among White MSM in Baltimore 2017–2023.

#### Powder Cocaine

The overall APP of powder cocaine use increased from 22% to 26% in DC, decreased from 16% to 12% in Baltimore, and increased from 12% to 23% in Philadelphia. Among Black MSM, APP of powder cocaine use increased in DC (17% to 25%) and Philadelphia (10% to 13%) but decreased in Baltimore (12% to 10%). Among White MSM, APP for cocaine increased modestly in DC (23% to 24%) and Baltimore (20% to 22%) and dramatically in Philadelphia (16% to 50%). Pairwise comparisons showed statistically significant changes over time for most periods, with the notable exception of stable use among Black MSM in Baltimore (2008–2011).

#### Crack Cocaine

The overall APP of crack cocaine use increased from 3% to 5% in DC, decreased from 12% to 10% in Baltimore, and remained stable at 6% in Philadelphia (2008–2023 comparison non-significant). Among Black MSM, APP of crack cocaine use increased in DC (2% to 8%) but decreased in Baltimore (12% to 10%) and Philadelphia (6% to 5%). Among White MSM, APP for crack cocaine remained consistently low (1% to 0%) in DC, decreased in Baltimore (12% to 10%), and increased in Philadelphia (5% to 8%). Pairwise comparisons confirmed statistically significant changes over time for most trends, with notable stability in Philadelphia (2008–2023), among White MSM in Baltimore (2011 and 2023 compared to 2008) and Philadelphia (2008–2011).

## Discussion

This study examined trends over the past 15 years (from 2008 to 2023) in stimulant use among MSM by race/ethnicity across three neighboring Mid-Atlantic metropolitan areas, each highly affected by substance use and HIV infection but with unique social, public health, and substance use contexts. Data revealed an overall increasing trend in stimulant use over the study period, as others have shown in different contexts [37], but with substantial variation across the region and by race/ethnicity.

Philadelphia saw major increases for all three drugs, while use prevalence was relatively stable in DC and changes in Baltimore were variable by race and socio-economic status. Overall increases were highest among White MSM, particularly in Philadelphia where predicted probability reached 50% for powder cocaine and 19% for methamphetamine. Stimulant use generally increased among Hispanic MSM, especially in Philadelphia. Use patterns were more variable among Black MSM, with increases for some drugs in some cities and decreases in others.

There was a strong increase in methamphetamine use prevalence in Philadelphia, particularly among White and Hispanic MSM, and more modest increases among White and Black MSM in Baltimore and Black MSM in DC. Powder cocaine use increased substantially in Philadelphia but increased with much less magnitude in DC and decreased in Baltimore. Crack cocaine use was less prevalent overall, and changes were less pronounced and more variable across cities. Initial prevalence was highest in Baltimore but decreased over time and there were small increases in DC and in Philadelphia. Across cities and drug types, socioeconomic marginalization (e.g., unhoused status and poverty) and living with HIV infection were consistently associated with higher odds of stimulant use.

These findings show that stimulant use prevalence among MSM in these Mid-Atlantic cities largely exceeded national averages for MSM in 2023 [38]. Methamphetamine use in Philadelphia was almost double the 2023 national average among MSM. Powder cocaine use in Philadelphia and DC was similar to national levels, while Baltimore was almost 50% lower. Crack cocaine use was notably higher in all three cities compared to national prevalence, and nearly three times higher in Baltimore.

These data provide a fuller understanding of substance use among MSM by characterizing stimulant use patterns and demonstrating variation across populations and geographies. Insights into stimulant use patterns enable providers and public health officials to better tailor resources and services while avoiding assumptions and stereotypes. While there are clear implications for addressing HIV risk and treatment adherence concerns associated with increased stimulant use [39], they also reinforce the need for effective stimulant treatment overall and those tailored for diversity among MSM [21, 25, 40]. The regional patterns, particularly the increases in methamphetamine use and steadily high prevalence of crack cocaine use in Baltimore, suggest a need for geographically tailored interventions. The variation in stimulant use by HIV status within the national data also suggests racial differences in stimulant use prevalence in these cities may also partially reflect the disproportionate prevalence of HIV among Black MSM.

### Mid-Atlantic Regional Context

These findings illustrate evolving patterns of stimulant use among MSM in the Mid-Atlantic region, an area with historically high rates of substance use, unique drug market features, high HIV prevalence among MSM, and persistent racial disparities [41]. Methamphetamine use has generally been low on the East Coast, but evidence of increasing prevalence among MSM in the Northeast [42] and Mid-Atlantic [43] suggests geographic spread within this population is occurring across our study cities.

The divergent patterns of cocaine and crack use we observed—increases in powdered cocaine coupled with stable or decreasing crack cocaine use—may reflect changing drug market factors including cost, quality, availability, and evolving polysubstance use preferences. While regional trends suggest overall increases in stimulant use, there were also divergent trajectories across racial and ethnic groups where increases in one stimulant coincided with decreases in another. For example, in DC, methamphetamine use among White MSM declined in earlier years but increased from 2017 to 2023, while powdered cocaine and crack use declined during the same period. Similarly, methamphetamine declines among Hispanic MSM in 2023 coincided with increases in powdered cocaine and crack.

#### Drug Market Shifts

Major drug supply changes occurred across the region during the study period that may help to explain these findings. DEA data indicates methamphetamine seizures in Pennsylvania increased by 1,500% between 2008 and 2023 [44], while recent major cocaine seizures at the Port of Philadelphia [45] reflect increased US trafficking of cocaine and methamphetamine that has driven down prices and increased drug availability and potency [46]. Simultaneously, fentanyl largely replaced heroin, opioid-stimulant polysubstance use became more widely recognized, overdose mortality soared—particularly stimulant-involved deaths [47, 48], and overall drug use patterns have evolved.

Methamphetamine, once historically scarce in the region, was reported by 8% of Baltimore PWID participants in 2024 [49] and 15% of Black MSM recruited through online platforms [43]. In DC, NHBS community stakeholders identified growing methamphetamine presence particularly during and after the COVID pandemic, accompanying changes in availability and cost [50] that may explain the increase in use observed in 2023. Methamphetamine injection has also been associated with recent HIV outbreaks in the region, including a cluster of linked HIV cases among an MSM methamphetamine-using sexual network in DC identified through molecular epidemiology [51], reinforcing the intersection of these trends with HIV transmission risk.

####  Intersection with HIV Response

The study period coincided with intensified culturally responsive HIV prevention efforts across all three cities, including community mobilization, biomedical prevention infrastructure expansion, and stigma reduction initiatives in response to high HIV incidence and recognized racial disparities among MSM [41]. In Baltimore, substantial improvements in new HIV diagnoses and care continuum outcomes were documented during the study period, particularly among Black MSM experiencing the highest burden of HIV infection [52]. Maryland also initiated programming to strengthen sexual health and diversity competence within behavioral health settings in response to recognized gaps in LGBTQ+-tailored services. It is plausible that these cultural competency efforts and HIV-focused strategies also influenced substance use behaviors and socialization patterns.

However, our findings—including elevated crack use, emerging methamphetamine trends, and increased odds of stimulant use among some racial/ethnic groups of MSM living with HIV—also underscore continued gaps. Since 2018, Philadelphia has been responding to an HIV outbreak among PWID, of which more than 1 in 5 are MSM/PWID [53]. Increased adjusted odds of methamphetamine and cocaine use among some racial/ethnic groups of MSM living with HIV in Philadelphia, combined with the negative impact of stimulant use on ART adherence [54], underscore the urgency of expanding LGBTQ+-affirming stimulant treatment programs as a necessary component of HIV prevention and care.

### Limitations

There are important limitations to consider. The 15-year data collection period encompasses major public health and socio-political developments that may have influenced stimulant use patterns. These include the COVID-19 pandemic, the synthetic opioid and polysubstance use overdose crisis, Affordable Care Act implementation and expanded health care access, and other policy changes. This analysis shows that local NHBS data is critical, as it helps identify city-specific and demographic trends that inform targeted local EHE initiatives, recognizing that the timing and impact of changes vary across communities and are often difficult to disentangle.

Data collection for NHBS is limited to MSM recruited from venues, which inherently misses those who do not socialize in public spaces with high MSM concentration. Because NHBS eligibility is limited to MSM who live in the same jurisdiction as data collection, it also cannot account for those who socialize outside of their home jurisdiction. Given the social patterns across the three cities represented in this analysis, it is important to note that the analyses would likely miss MSM from one city (e.g., Baltimore) who primarily attend events and venues in neighboring cities (e.g., DC and Philadelphia). Also, the MSM recruited through venue-based sampling may have changed as LGBTQ+ venues have declined over time and internet sites and apps have largely supplanted physical venues for partner seeking [55].

All data are based on self-report and may be influenced by social desirability. This could affect disclosure in both directions. Participants may be less willing to discuss drug use due to stigma, despite substantial efforts since the beginning of NHBS to foster trust in the projects and build rapport with communities and individual participants. However, it is also possible that shifting norms related to drug use during the study period, notably affected by policy changes around cannabis in all jurisdictions and decriminalization efforts in Baltimore, may have increased willingness to disclose.

The data are not weighted to account for potential venue selection bias or variability across venues beyond type, all of which may also have changed over time, and these analyses do not directly test associations with HIV transmission risk. Finally, the data are all cross-sectional, and it is possible that there is overlap in study participants over time – although assessments suggest this is relatively minimal. Still, the best interpretation of the data are as serial point-in-time assessments of the population of MSM who live in each respective city and attend MSM venues in their city of residence.

### Public Health Implications

The U.S. is at a critical juncture in the HIV epidemic. Although each of these cities have made substantial progress in meeting ‘Ending the HIV Epidemic’ goals, MSM continue to account for the majority of HIV infections, racial and ethnic disparities remain entrenched, and underlying social determinants persist. Furthermore, the evolving nature of substance use in the U.S. has reinforced the critical need for attention to stimulant use even as public health efforts prioritize opioid overdose prevention strategies. Recently released clinical practice guidelines for stimulant use disorder treatment [13] highlight the urgency of developing effective therapeutics [56, 57] - as there are currently no FDA-approved pharmacological treatments for stimulants - and the need for appropriate interventions for sexual and gender minority populations. This study suggests that addressing barriers to stimulant-related harm reduction and treatment for MSM will require a syndemics-oriented approach to culturally-competent and LGBTQ+-affirming services that address individual-level needs as well as structural factors contributing to health disparities [58, 59].

The substantial geographic and demographic variation observed across these neighboring Mid-Atlantic cities underscores the need for locally tailored strategies that account for unique contextual factors, community characteristics, and behavioral patterns rather than a uniform approach [41]. This study also demonstrates the unique value of a standardized national HIV behavioral surveillance system. By using identical protocols and rigorous population-based sampling methods to produce locally representative socio-behavioral data, NHBS enables meaningful comparisons across cities and regions while also providing detailed information for local and national level public health planning.

Sustaining progress towards EHE goals for MSM will require continued investment in evidence-based HIV prevention and care services, culturally appropriate harm reduction and treatment approaches that can address health risks associated with stimulant use, social supports and safety nets that mitigate the implications of social instability for substance use and HIV transmission risk, and the vital data systems such as NHBS that enable local and national insights about public health needs among marginalized populations that would otherwise be missed.

## Supplementary Information

Below is the link to the electronic supplementary material.

Supplementary Material 1

## Declarations

### Research involving Human and animal participants

For Washington, DC, the DC Department of Health IRB and the George Washington University IRB reviewed and approved NHBS protocols. For Philadelphia, the City of Philadelphia Department of Public Health IRB reviewed and approved NHBS protocols. For Baltimore, the NHBS protocol and all local materials were approved by Institutional Review Boards at the Johns Hopkins Bloomberg School of Public Health and the Maryland Department of Health.

## References

[1] Fauci AS, Redfield RR, Sigounas G, Weahkee MD, Giroir BP. Ending the HIV Epidemic: A Plan for the United States. Jama Mar. 2019;5(9):844–5. 10.1001/jama.2019.1343 30730529

[2] Giroir BP. The time is now to end the HIV epidemic. Am J Public Health. 2020;110(1):22–4. 31725312 10.2105/AJPH.2019.305380 PMC6893354

[3] Bleichrodt AM, Okano JT, Fung ICH, Chowell G, Blower S. The future of HIV: challenges in meeting the 2030 Ending the HIV Epidemic in the US (EHE) reduction goal. Aids May. 2025;1(6):708–18. 10.1097/QAD.0000000000004122 PMC11968241 39832182

[4] Centers for Disease Control and Prevention. Preexposure prophylaxis for the prevention of HIV infection in the United States -- 2021 update: a clinical practice guideline 2021.

[5] Babel RA, Wang P, Alessi EJ, Raymond HF, Wei C. Stigma, HIV risk, and access to HIV prevention and treatment services among men who have sex with men (MSM) in the United States: a scoping review. AIDS Behav. 2021;25(11):3574–604. 33866444 10.1007/s10461-021-03262-4 PMC8053369

[6] Beyrer C, Sullivan P, Sanchez J, et al. The increase in global HIV epidemics in MSM. Aids Nov. 2013;13(17):2665–78. 10.1097/01.aids.0000432449.30239.fe 23842129

[7] National Academies of Sciences, Engineering, and Medicine, Division of Behavioral and Social Sciences and Education; Committee on Population; Committee on Understanding the Well-Being of Sexual and Gender Diverse Populations. ; White J, Sepúlveda MJ, Patterson CJ, editors. Understanding the Well-Being of LGBTQI+ Populations. Washington (DC): National Academies Press (US); 2020 Oct 21. Available from: https://www.ncbi.nlm.nih.gov/books/NBK563325/doi: 33104312

[8] Compton WM, Jones CM. Substance Use among Men Who Have Sex with Men. N Engl J Med Jul. 2021;22(4):352–6. 10.1056/NEJMra2033007 PMC9169429 34289278

[9] Goldshear JL, Westmoreland DA, Carrico AW, Grov C. Drug use typology, demographic covariates, and associations with condomless anal sex: a latent class analysis among a U.S. national cohort of men who have sex with men. Int J Drug Policy. 2023;112:103949. 36587507 10.1016/j.drugpo.2022.103949 PMC9975079

[10] Philbin MM, Greene ER, Martins SS, LaBossier NJ, Mauro PM. Medical, nonmedical, and illegal stimulant use by sexual identity and gender. Am J Prev Med. 2020;59(5):686–96. 32981768 10.1016/j.amepre.2020.05.025 PMC7577928

[11] Centers for Disease Control and Prevention. HIV infection risk, prevention, and testing behaviors among men who have sex with men-National HIV Behavioral Surveillance, 20 US Cities, 2014. HIV Surveillance Special Rep. 2016;15(4):2009–13.

[12] Maxwell S, Shahmanesh M, Gafos M. Chemsex behaviours among men who have sex with men: A systematic review of the literature. Int J Drug Policy Jan. 2019;63:74–89. 10.1016/j.drugpo.2018.11.014 30513473

[13] The ASAM/AAAP Clinical Practice Guideline on the Management of Stimulant Use Disorder. J Addict Med May-Jun. 2024;01(1S Suppl 1):1–56. 10.1097/ADM.0000000000001299 PMC11105801 38669101

[14] Farrell M, Martin NK, Stockings E, et al. Responding to global stimulant use: challenges and opportunities. Lancet Nov. 2019;2(10209):1652–67. 10.1016/S0140-6736(19)32230-5 PMC6924572 31668409

[15] Duflou J. Psychostimulant use disorder and the heart. Addiction Jan. 2020;115(1):175–83. 10.1111/add.14713 31321853

[16] Lappin JM, Sara GE. Psychostimulant use and the brain. Addiction Nov. 2019;114(11):2065–77. 10.1111/add.14708 31321819

[17] Park JN, Rashidi E, Foti K, Zoorob M, Sherman S, Alexander GC. Fentanyl and fentanyl analogs in the illicit stimulant supply: Results from U.S. drug seizure data, 2011–2016. Drug Alcohol Depend Jan. 2021;1:218:108416. 10.1016/j.drugalcdep.2020.108416 PMC7751390 33278761

[18] Mayer KH, Nelson L, Hightow-Weidman L, et al. The persistent and evolving HIV epidemic in American men who have sex with men. Lancet Mar. 2021;20(10279):1116–26. 10.1016/S0140-6736(21)00321-4 PMC9639667 33617771

[19] Ivey K, Bernstein KT, Kirkcaldy RD, et al. Chemsex Drug Use among a National Sample of Sexually Active Men who have Sex with Men, - American Men’s Internet Survey, 2017–2020. Subst Use Misuse. 2023;58(5):728–34. 36872623 10.1080/10826084.2023.2184207 PMC10167950

[20] Shoptaw S, Li MJ, Javanbakht M, Ragsdale A, Goodman-Meza D, Gorbach PM. Frequency of reported methamphetamine use linked to prevalence of clinical conditions, sexual risk behaviors, and social adversity in diverse men who have sex with men in Los Angeles. Drug Alcohol Depend. 2022;232:109320. 35093681 10.1016/j.drugalcdep.2022.109320 PMC8885921

[21] Viera A, Sosnowy CD, van den Berg JJ, et al. Substance Use Treatment Engagement among Men Who Have Sex with Men Who Use Stimulants in the Northeastern United States. Subst Use Misuse. 2022;57(4):595–602. 35068332 10.1080/10826084.2022.2026965 PMC9343172

[22] Hojilla JC, Vlahov D, Glidden DV, et al. Skating on thin ice: stimulant use and sub-optimal adherence to HIV pre-exposure prophylaxis. J Int AIDS Soc. 2018;21(3):e25103. 29577616 10.1002/jia2.25103 PMC5867332

[23] Carrico AW, Woolf-King SE, Neilands TB, Dilworth SE, Johnson MO. Stimulant use and HIV disease management among men in same-sex relationships. Drug Alcohol Depend. 2014;139:174–7. 24726318 10.1016/j.drugalcdep.2014.03.025 PMC4048569

[24] Viera A, van den Berg JJ, Sosnowy CD, et al. Barriers and facilitators to HIV pre-exposure prophylaxis uptake among men who have sex with men who use stimulants: a qualitative study. AIDS Behav. 2022;26(9):3016–28. 35303188 10.1007/s10461-022-03633-5 PMC9378498

[25] Noor SW, Hart TA, Okafor CN, et al. Staying or moving: Results of a latent transition analysis examining intra-individual stability of recreational substance use among MSM in the Multicenter AIDS Cohort Study from 2004 to 2016. Drug Alcohol Depend Mar. 2021;1:220:108516. 10.1016/j.drugalcdep.2021.108516 PMC7901540 33485009

[26] Thiede H, Valleroy LA, MacKellar DA, et al. Regional patterns and correlates of substance use among young men who have sex with men in 7 US urban areas. Am J Public Health. 2003;93(11):1915–21. 14600066 10.2105/ajph.93.11.1915 PMC1448076

[27] Feinstein BA, Moody RL, John SA, Parsons JT, Mustanski B. A three-city comparison of drug use and drug use before sex among young men who have sex with men in the United States. J Gay Lesbian Soc Serv. 2018;30(1):82–101. 30381785 10.1080/10538720.2018.1408519 PMC6205241

[28] Hirshfield S, Remien RH, Humberstone M, Walavalkar I, Chiasson MA. Substance use and high-risk sex among men who have sex with men: a national online study in the USA. AIDS Care. 2004;16(8):1036–47. 15511735 10.1080/09540120412331292525

[29] Westmoreland DA, Carrico AW, Goodwin RD, Pantalone DW, Nash D, Grov C. Higher and higher? Drug and alcohol use and misuse among HIV-vulnerable men, trans men, and trans women who have sex with men in the United States. Subst Use Misuse. 2021;56(1):111–22. 33153358 10.1080/10826084.2020.1843057 PMC8218329

[30] Wejnert C, Hess KL, Rose CE, et al. Age-Specific Race and Ethnicity Disparities in HIV Infection and Awareness Among Men Who Have Sex With Men–20 US Cities, 2008–2014. J Infect Dis Mar. 2016;1(5):776–83. 10.1093/infdis/jiv500 PMC6281353 26486637

[31] Gallagher KM, Sullivan PS, Lansky A, Onorato IM. Behavioral surveillance among people at risk for HIV infection in the U.S.: the National HIV Behavioral Surveillance System. Public Health Rep. 2007;122(Suppl 1):32–8. 17354525 10.1177/00333549071220S106 PMC1804113

[32] Kanny D, Broz D, Finlayson T, Lee K, Sionean C, Wejnert C. A Key Comprehensive System for Biobehavioral Surveillance of Populations Disproportionately Affected by HIV (National HIV Behavioral Surveillance): Cross-sectional Survey Study. JMIR public health surveillance Nov. 2022;15(11):e39053. 10.2196/39053 PMC9709677 36378503

[33] US Department of Justice. Drug Enforcement Agency. 2020 National Drug Threat Assessment. 2021. Available https://www.dea.gov/sites/default/files/2021-02/DIR-008-21%202020%20National%20Drug%20Threat%20Assessment_WEB.pdf. Accessed January 18, 2026.

[34] Washington/Baltimore HIDTA, High Intensity Drug Trafficking Area. *Annual Report* 2025. Available https://www.hidta.org/wp-content/uploads/2025/07/WB-HIDTA-2024-Annual-Report-Web.pdf Accessed January 18, 2026.

[35] Liberty Mid-Atlantic HIDTA, Regional Drug Trends. 2026. Accessed January 18, 2026. https://lmahidta.org/regional-drug-threat/#1513218835842-75c151f2-40bf

[36] MacKellar DA, Gallagher KM, Finlayson T, Sanchez T, Lansky A, Sullivan PS. Surveillance of HIV risk and prevention behaviors of men who have sex with men–a national application of venue-based, time-space sampling. Public Health Rep. 2007;122(Suppl 1):39–47. 17354526 10.1177/00333549071220S107 PMC1804106

[37] Coyer L, Boyd A, Davidovich U, van Bilsen WPH, Prins M, Matser A. Increase in recreational drug use between 2008 and 2018: results from a prospective cohort study among HIV-negative men who have sex with men. Addiction. 2022;117(3):656–65. 34402120 10.1111/add.15666 PMC9292014

[38] Centers for Disease Control and Prevention. *HIV Surveillance Special Report: HIV Infection Risk, Prevention, and Testing Behaviors Among Men Who Have Sex With Men—National HIV Behavioral Surveillance, 19 U.S. Cities, 2023*. https://stacks.cdc.gov/view/cdc/162349 Accessed January 30, 2026.

[39] Lim SH, Ostrow D, Stall R, et al. Changes in stimulant drug use over time in the MACS: evidence for resilience against stimulant drug use among men who have sex with men. AIDS Behav. 2012;16(1):151–8. 21191644 10.1007/s10461-010-9866-x PMC3133874

[40] Mimiaga MJ, Reisner SL, Fontaine YM, et al. Walking the line: stimulant use during sex and HIV risk behavior among Black urban MSM. Drug Alcohol Depend Jul. 2010;1(1–2):30–7. 10.1016/j.drugalcdep.2010.01.017 PMC3947405 20334986

[41] German D, Brady K, Kuo I, Characteristics of Black Men Who Have Sex With Men in Baltimore, Philadelphia, and, Washington DC et al. Geographic Diversity in Socio-Demographics and HIV Transmission Risk. *J Acquir Immune Defic Syndr.* Jul 1 2017;75 Suppl 3(Suppl 3):S296-s308. 10.1097/QAI.0000000000001425 PMC5499158 28604431

[42] Rivera AV, Harriman G, Carrillo SA, Braunstein SL. Trends in methamphetamine use among men who have sex with men in New York City, 2004–2017. AIDS Behav. 2021;25(4):1210–8. 33185774 10.1007/s10461-020-03097-5 PMC8190824

[43] Jennings JM, Wagner J, Tilchin C, et al. Methamphetamine Use, Syphilis, and Specific Online Sex Partner Meeting Venues Are Associated With HIV Status Among Urban Black Gay and Bisexual Men Who Have Sex Men. Sex Transm Dis Aug. 2021;1(8s):S32–9. 10.1097/OLQ.0000000000001452 PMC8284367 33967238

[44] U.S. Drug Enforcement Administration Diversion Control Division. *State counts for methamphetamine: 2008–2023.* 2025. Available https://www.nflis.deadiversion.usdoj.gov/ Accessed January 15, 2026.

[45] U.S. Customs and Border Protection. U.S. Customs and Border Protection Seizes over 17.5 Tons of Cocaine in Philadelphia. Accessed January 15, 2026. Available https://www.cbp.gov/newsroom/national-media-release/us-customs-and-border-protection-seizes-over-175-tons-cocaine

[46] Whelan A. Cocaine and methamphetamine are on the rise in Philly, even as the opioid crisis continues. *Inquirer.com.* October 1, 2019. Available Accessed January 15, 2026. https://www.inquirer.com/health/opioid-addiction/philadelphia-pennsylvania-meth-cocaine-stimulant-use-rising-20191001.htm

[47] City of Philadelphia. Philadelphia Records More than 1,400 Overdose Deaths in 2022; Deaths among Black Residents Rose Nearly 20%. Available Accessed January 15, 2026. https://www.phila.gov/2023-10-02-philadelphia-records-more-than-1400-overdose-deaths-in-2022-deaths-among-black-residents-rose-nearly-20/

[48] Tanz LJ, Miller KD, Dinwiddie AT, et al. Drug Overdose Deaths Involving Stimulants ― United States, January 2018–June 2024. MMWR Morb Mortal Wkly Rep 2025;74:491–499. 10.15585/mmwr.mm7432a1 PMC12393691 40875496

[49] German D, McMahon M, Ashburn K, Gribbin M, Flynn C. HIV Infection Risk, Prevention, and Testing Behaviors among People Who Inject Drugs —Baltimore HIV Behavioral Surveillance, 2023. Baltimore HIV Behavioral Surveillance Special Report. 2025.

[50] White House Office of National Drug Control Policy. Methamphetamine Plan Implementation Report. April 2023. Available Accessed January 15, 2026. https://www.whitehouse.gov/wp-content/uploads/2025/03/ONDCP-Methamphetamine-Plan-Implementation-Report_April-2023.pdf

[51] Perez SM, Panneer N, France AM, et al. Clusters of Rapid HIV Transmission Among Gay, Bisexual, and Other Men Who Have Sex with Men - United States, 2018–2021. MMWR Morb Mortal Wkly Rep Sep. 2022;23(38):1201–6. 10.15585/mmwr.mm7138a1 PMC9531569 36136909

[52] German D, Gribbin M, Spencer L, Burrell J Jr., Kennedy K, Flynn C. Racial disparities in HIV prevalence, testing, and care among men who have sex with men in Baltimore from 2008–2023: Fifteen years of HIV behavioral surveillance. *AIDS & Behavior.* 2026; 2026 Feb 19. 10.1007/s10461-026-05031-7. Epub ahead of print. PMID: 41712052. PMC13202490 41712052

[53] Philadelphia Department of Public Health. *Surveillance Report, 2023.* Philadelphia, PA December 2024.

[54] Socias ME, Milloy MJ. Substance Use and Adherence to Antiretroviral Therapy: What Is Known and What Is Unknown. Curr Infect Dis Rep Jul. 2018;31(9):36. 10.1007/s11908-018-0636-7 PMC6375705 30066113

[55] Paz-Bailey G, Hoots BE, Xia M, Finlayson T, Prejean J, Purcell DW. Trends in Internet Use Among Men Who Have Sex With Men in the United States. J Acquir Immune Defic Syndr Jul. 2017;1(Suppl 3):S288–95. 10.1097/QAI.0000000000001404 PMC5871925 28604430

[56] Ronsley C, Nolan S, Knight R, et al. Treatment of stimulant use disorder: a systematic review of reviews. PLoS One. 2020;15(6):e0234809. 32555667 10.1371/journal.pone.0234809 PMC7302911

[57] Young EJ, Radnai L, Prikhodko V, Miller CA. Novel therapeutics in development for the treatment of stimulant-use disorder. Curr Opin Neurobiol. 2024;87:102898. 39096558 10.1016/j.conb.2024.102898 PMC13034452

[58] Bourne A, Weatherburn P. Substance use among men who have sex with men: patterns, motivations, impacts and intervention development need. Sex Transm Infect. 2017;93(5):342–6. 28400466 10.1136/sextrans-2016-052674

[59] Rosner B, Neicun J, Yang JC, Roman-Urrestarazu A. Substance use among sexual minorities in the US - linked to inequalities and unmet need for mental health treatment? Results from the National Survey on Drug Use and Health (NSDUH). Journal psychiatric Res Mar. 2021;135:107–18. 10.1016/j.jpsychires.2020.12.023 33472121

