# Supplementary material for: Changes in Stimulant Use Over Time Among Men Who Have Sex with Men in Philadelphia, Baltimore, and Washington, DC, 2008–2023
Source: AIDS Behav. 2026 Mar 31;30(9):2694–707. doi: 10.1007/s10461-026-05098-2 (PMC13202489; doi:10.1007/s10461-026-05098-2)
Supplement: Supplementary file 1 [file 10461_2026_5098_MOESM1_ESM.docx]

**Supplemental Table 1. Prevalence of past year use of stimulant drugs among MSM enrolled in NHBS in Washington DC, Baltimore, and Philadelphia, 2008-2023.**

|  | **Washington, DC** | | | | **Baltimore, MD** | | | | **Philadelphia, PA** | | | |
| --- | --- | --- | --- | --- | --- | --- | --- | --- | --- | --- | --- | --- |
|  | **Black MSM**  **n (%)** | **White MSM**  **n (%)** | **Hispanic MSM**  **n (%)** | **Total MSM**  **N (%)** | **Black MSM**  **n (%)** | **White MSM**  **n (%)** | **Hispanic MSM**  **n (%)** | **Total MSM**  **N (%)** | **Black MSM**  **(%)** | **White MSM**  **(%)** | **Hispanic MSM**  **(%)** | **Total MSM**  **N (%)** |
| **Methamphetamine** | | | | | | | | | | | | |
| **2008** | 7/158 (4.4) | 23/241 (9.5) | 3/24 (12.5) | 33/423 (7.8) | 4/323 (1.2) | 5/116 (4.3) | 0/21 (0) | 9/460 (2.0) | 5/182 (2.8) | 10/305 (3.3) | 1/52 (1.9) | 16/539 (3.0) |
| **2011** | 9/150 (6.0) | 12/237 (5.1) | 2/73 (2.7) | 23/460 (5.0) | 5/348 (1.4) | 2/64 (3.1) | 0/10 (0) | 7/422 (1.7) | 5/314 (1.6) | 6/143 (4.2) | 0/63 (0) | 11/520 (2.1) |
| **2014** | 21/213 (9.9) | 9/191 (4.7) | 6/65 (9.2) | 36/469 (7.7) | 1/326 (0.3) | 8/111 (7.2) | 0/24 (0) | 9/461 (2.0) | 14/326 (4.3) | 17/170 (10.0) | 4/101 (4.0) | 35/597 (5.9) |
| **2017** | 10/218 (4.6) | 8/148 (5.4) | 9/81 (11.1) | 27/447 (6.0) | 6/281 (2.1) | 5/65 (7.7) | 0/24 (0) | 11/370 (3.0) | 10/369 (2.7) | 14/69 (20.3) | 5/69 (7.3) | 29/507 (5.7) |
| **2023** | 5/65 (7.7) | 8/76 (10.5) | 1/26 (3.9) | 14/167 (8.4) | 10/212 (4.7) | 4/54 (7.4) | 0/13 (0) | 14/279 (5.0) | 15/230 (6.5) | 13/68 (19.1) | 7/54 (13.0) | 35/352 (9.9) |
| **Powdered Cocaine** | | | | | | | | | | | | |
| **2008** | 29/158 (18.4) | 50/241 (20.8) | 7/24 (29.2) | 86/423 (20.3) | 45/323 (13.9) | 24/116 (20.7) | 2/21 (9.5) | 71/460 (15.4) | 14/182 (7.7) | 44/305 (14.4) | 5/52 (9.6) | 63/539 (11.7) |
| **2011** | 18/150 (12.0) | 43/237 (18.1) | 5/73 (6.9) | 66/460 (14.4) | 39/348 (11.2) | 10/64 (15.6) | 0/10 (0) | 49/422 (11.6) | 41/314 (13.1) | 19/143 (13.3) | 6/63 (9.5) | 66/520 (12.7) |
| **2014** | 35/213 (16.4) | 40/191 (20.9) | 12/65 (18.5) | 87/469 (18.6) | 28/326 (8.6) | 25/111 (22.5) | 3/24 (12.5) | 56/461 (12.2) | 52/326 (16.0) | 37/170 (21.8) | 20/101 (19.8) | 109/597 (18.3) |
| **2017** | 37/218 (17.0) | 35/148 (23.7) | 12/81 (14.8) | 84/447 (18.8) | 25/281 (8.9) | 12/65 (18.5) | 3/24 (12.5) | 40/370 (10.8) | 47/369 (12.7) | 15/69 (21.7) | 13/69 (18.8) | 75/507 (14.8) |
| **2023** | 17/65 (26.2) | 17/76 (22.4) | 5/26 (19.2) | 39/167 (23.4) | 19/212 (9.0) | 13/54 (24.1) | 3/13 (23.1) | 35/279 (12.5) | 34/230 (14.8) | 28/68 (41.2) | 14/54 (25.9) | 76/352 (21.6) |
| **Crack Cocaine** | | | | | | | | | | | | |
| **2008** | 3/158 (1.9) | 6/241 (2.5) | 1/24 (4.2) | 10/423 (2.4) | 45/323 (13.9) | 8/116 (6.9) | 1/21 (4.8) | 54/460 (11.7) | 10/182 (5.5) | 13/305 (4.3) | 2/52 (3.9) | 25/539 (4.6) |
| **2011** | 2/150 (1.3) | 9/237 (3.8) | 0/73 (0.0) | 11/460 (2.4) | 48/348 (13.8) | 6/64 (9.4) | 1/10 (10.0) | 55/422 (13.0) | 11/314 (3.5) | 6/143 (4.2) | 2/63 (3.2) | 19/520 (3.7) |
| **2014** | 6/213 (2.8) | 6/191 (3.1) | 1/65 (1.5) | 13/469 (2.8) | 28/326 (8.6) | 7/111 (6.3) | 1/24 (4.2) | 36/461 (7.8) | 31/326 (9.5) | 15/170 (8.8) | 5/101 (5.0) | 51/597 (8.5) |
| **2017** | 9/218 (4.1) | 2/148 (1.4) | 4/81 (4.9) | 15/447 (3.4) | 24/281 (8.5) | 10/65 (15.4) | 2/24 (8.3) | 36/370 (9.7) | 18/369 (4.9) | 11/69 (15.9) | 2/69 (2.9) | 31/507 (6.1) |
| **2023** | 3/65 (4.6) | 0/76 (0.0) | 3/26 (11.5) | 6/167 (3.6) | 22/212 (10.4) | 9/54 (16.7) | 0/13 (0) | 31/279 (11.1) | 17/230 (7.4) | 6/68 (8.8) | 5/54 (9.3) | 28/352 (8.0) |

**Supplemental table 2. Average predicted probability of stimulant use by drug among MSM enrolled in NHBS in Washington DC, Baltimore, and Philadelphia, 2008-2023 (95% bootstrap confidence limits)**

|  | 2008 | 2011 | 2014 | 2017 | 2023 |
| --- | --- | --- | --- | --- | --- |
| **WASHINGTON DC** |  |  |  |  |  |
| Methamphetamine | 0.099 (0.061, 0.143) | 0.060 (0.039, 0.089) | 0.080 (0.056, 0.106) | 0.047 (0.032, 0.064) | 0.094 (0.052, 0.142) |
| Powdered cocaine | 0.219 (0.172, 0.271) | 0.148 (0.118, 0.179) | 0.174 (0.141, 0.210) | 0.202 (0.167, 0.239) | 0.264 (0.195, 0.334) |
| Crack cocaine | 0.029 (0.011, 0.050) | 0.031 (0.016, 0.049) | 0.023 (0.012, 0.039) | 0.023 (0.012, 0.036) | 0.050 (0.014, 0.104) |
| **BALTIMORE** |  |  |  |  |  |
| Methamphetamine | 0.019 (0.010, 0.032) | 0.024 (0.009, 0.040) | 0.018 (0.007, 0.033) | 0.026 (0.013, 0.044) | 0.049 (0.022, 0.086) |
| Powdered cocaine | 0.155 (0.119, 0.196) | 0.131 (0.097, 0.165) | 0.133 (0.099, 0.168) | 0.125 (0.095, 0.161) | 0.120 (0.077, 0.174) |
| Crack cocaine | 0.116 (0.079, 0.154) | 0.153 (0.116, 0.186) | 0.090 (0.062, 0.122) | 0.103 (0.074, 0.138) | 0.095 (0.065, 0.130) |
| **PHILADELPHIA** |  |  |  |  |  |
| Methamphetamine | 0.039 (0.018, 0.068) | 0.028 (0.011, 0.047) | 0.053 (0.036, 0.071) | 0.069 (0.044, 0.100) | 0.087 (0.060, 0.115) |
| Powdered cocaine | 0.117 (0.081, 0.157) | 0.132 (0.097, 0.169) | 0.185 (0.154, 0.216) | 0.155 (0.120, 0.195) | 0.228 (0.181, 0.274) |
| Crack cocaine | 0.055 (0.030, 0.089) | 0.043 (0.024, 0.066) | 0.075 (0.054, 0.095) | 0.072 (0.048, 0.104) | 0.057 (0.037, 0.078) |

**Supplemental table 3. Average predicted probability of stimulant use by drug and by race/ethnicity among MSM enrolled in NHBS in Washington DC, Baltimore, and Philadelphia, 2008-2023 (95% bootstrap confidence limits)**

| **WASHINGTON DC** | 2008 | 2011 | 2014 | 2017 | 2023 |
| --- | --- | --- | --- | --- | --- |
| **Black MSM** |  |  |  |  |  |
| Methamphetamine | 0.167 (0.017, 0.204) | 0.064 (0.025, 0.111) | 0.107 (0.062, 0.166) | 0.033 (0.015, 0.061) | 0.051 (0.020, 0.231) |
| Powdered Cocaine | 0.171 (0.111, 0.294) | 0.124 (0.072, 0.190) | 0.158 (0.107, 0.218) | 0.174 (0.114, 0.232) | 0.246 (0.125, 0.372) |
| Crack Cocaine | 0.016 (0.004, 0.090) | 0.006 (0.002, 0.039) | 0.025 (0.006, 0.051) | 0.030 (0.009, 0.059) | 0.076 (0.018, 0.180) |
| **White MSM** |  |  |  |  |  |
| Methamphetamine | 0.094 (0.053, 0.138) | 0.056 (0.030, 0.094) | 0.047 (0.019, 0.081) | 0.061 (0.029, 0.114) | 0.127 (0.052, 0.218) |
| Powdered Cocaine | 0.233 (0.168, 0.302) | 0.175 (0.130, 0.229) | 0.196 (0.143, 0.260) | 0.252 (0.143, 0.260) | 0.237 (0.144, 0.350) |
| Crack Cocaine | 0.012 (0.004, 0.032) | 0.043 (0.016, 0.082) | 0.028 (0.009, 0.054) | 0.026 (0.016, 0.038) | -- |
| **BALTIMORE** | 2008 | 2011 | 2014 | 2017 | 2023 |
| **Black MSM** |  |  |  |  |  |
| Methamphetamine | 0.010 (0.002, 0.022) | 0.015 (0.003, 0.036) | 0.003 (0.001, 0.011) | 0.015 (0.004, 0.033) | 0.050 (0.016, 0.114) |
| Powdered Cocaine | 0.123 (0.088, 0.163) | 0.123 (0.086, 0.163) | 0.091 (0.055, 0.134) | 0.097 (0.061, 0.136) | 0.104 (0.053, 0.179) |
| Crack Cocaine | 0.116 (0.085, 0.151) | 0.162 (0.119, 0.214) | 0.099 (0.067, 0.135) | 0.085 (0.052, 0.126) | 0.102 (0.066, 0.157) |
| **White MSM** |  |  |  |  |  |
| Methamphetamine | 0.040 (0.010, 0.088) | 0.024 (0.009, 0.090) | 0.087 (0.031, 0.157) | 0.058 (0.015, 0.138) | 0.065 (0.013, 0.130) |
| Powdered Cocaine | 0.204 (0.132, 0.308) | 0.153 (0.068, 0.255) | 0.239 (0.149, 0.333) | 0.180 (0.097, 0.298) | 0.218 (0.121, 0.355) |
| Crack Cocaine | 0.118 (0.036, 0.219) | 0.095 (0.036, 0.203) | 0.076 (0.023, 0.148) | 0.117 (0.057, 0.213) | 0.097 (0.059, 0.212) |
| **PHILADELPHIA** | 2008 | 2011 | 2014 | 2017 | 2023 |
| **Black MSM** |  |  |  |  |  |
| Methamphetamine | 0.044 (0.010, 0.126) | 0.030 (0.006, 0.080) | 0.038 (0.018, 0.064) | 0.033 (0.015, 0.058) | 0.047 (0.023, 0.075) |
| Powdered Cocaine | 0.104 (0.043, 0.179) | 0.133 (0.094, 0.179) | 0.162 (0.122, 0.202) | 0.124 (0.092, 0.159) | 0.126 (0.087, 0.174) |
| Crack Cocaine | 0.055 (0.020, 0.108) | 0.040 (0.019, 0.071) | 0.088 (0.059, 0.119) | 0.069 (0.041, 0.102) | 0.048 (0.026, 0.072) |
| **White MSM** |  |  |  |  |  |
| Methamphetamine | 0.044 (0.020, 0.075) | 0.040 (0.009, 0.083) | 0.104 (0.061, 0.163) | 0.179 (0.093, 0.282) | 0.150 (0.080, 0.234) |
| Powdered Cocaine | 0.157 (0.114, 0.205) | 0.139 (0.074, 0.211) | 0.223 (0.160, 0.296) | 0.204 (0.160, 0.296) | 0.496 (0.367, 0.628) |
| Crack Cocaine | 0.053 (0.028, 0.081) | 0.066 (0.018, 0.131) | 0.081 (0.045, 0.143) | 0.129 (0.056, 0.228) | 0.081 (0.014, 0.162) |

**Supplemental table 4. Non-significant pairwise comparisons for predicted probabilities of stimulant use across time by city and by race/ethnicity by city among MSM NHBS participants in DC, Baltimore, and Philadelphia 2008-2023.**

| **Group** | **City** | **Methamphetamine** | **Powdered Cocaine** | **Crack Cocaine** |
| --- | --- | --- | --- | --- |
| **Overall** | DC | 2008-2023 (0.969) | - | 2014-2017 (0.979) |
|  | Baltimore | 2008-2014 (0.845) | - |  |
|  | Philadelphia | - | - | 2008-2023 (0.946)  2014-2017 (0.046) |
| **Black MSM** | DC | - | - | - |
|  | Baltimore | 2011-2017 (0.837) | 2008-2011 (1.00) | - |
|  | Philadelphia | 2008-2023 (0.327)  2011-2014 (0.290)  2011-2017 (0.181) | 2017-2023 (0.752) | - |
| **White MSM** | DC | - | - |  |
|  | Baltimore | 2017-2023 (1.00) | - | 2008-2011 (0.346)  2008-2023 (0.568) |
|  | Philadelphia | - | - | - |

*All other pairwise comparisons were statistically significant at p<0.05*
